# The regulatory effect of Tau protein on polymerization of MCF7 microtubules *in vitro*

**DOI:** 10.1016/j.bbrep.2018.12.010

**Published:** 2019-01-09

**Authors:** Mitra Shojania Feizabadi, Marcos A.V. Hernandez, Jane B. Breslin, Ibukunoluwa I. Akintola

**Affiliations:** Department of Physics, Seton Hall University, 400 South Orange Ave., South Orange, NJ 07079, USA

**Keywords:** Microtubule, MCF7 microtubule, Tau protein, Electrostatic specification, Electro-orientation, Tubulin isotype

## Abstract

Growing evidence continues to point toward the critical role of beta tubulin isotypes in regulating some intracellular functions. Changes that were observed in the microtubules’ intrinsic dynamics, the way they interact with some chemotherapeutic agents, or differences on translocation specifications of some molecular motors along microtubules, were associated to their structural uniqueness in terms of beta tubulin isotype distributions. These findings suggest that the effects of microtubule associated proteins (MAPs) may also vary on structurally different microtubules. Among different microtubule associated proteins, Tau proteins, which are known as neuronal MAPs, bind to beta tubulin, stabilize microtubules, and consequently promote their polymerizations.

In this study, in a set of well controlled experiments, the direct effect of Tau proteins on the polymerization of two structurally different microtubules, porcine brain and breast cancer (MCF7), were tested and compared. Remarkably, we found that in contrast with the promoted effect of Tau proteins on brain microtubules’ polymerization, MCF7 expressed a demoted polymerization while interacting with Tau proteins. This finding can potentially be a novel insight into the mechanism of drug resistance in some breast cancer cells.

It has been reported that microtubules show destabilizing behavior in some MCF7 cells with overexpression of Tau protein when treated with a microtubules’ stabilizing agent, Taxol. This behavior has been classified by others as drug resistance, but it may instead be potentially caused by a competition between the destabilizing effect of the Tau protein and the stabilizing effect of the drug on MCF7 microtubules. Also, we quantified the polarization coefficient of MCF7 microtubules in the presence and absence of Tau proteins by the electro-orientation method and compared the values. The two significantly different values obtained can possibly be one factor considered to explain the effect of Tau proteins on the polymerization of MCF7 microtubules.

## Highlights

• MCF7 microtubules express slow and stable polymerization behavior.
• Tau-MCF7 microtubules express demoted polymerization behavior.
• Tau-MCF7 polymerization can possibly be explained by electrostatic specifications.

## 1 Introduction

Microtubules, one of the intra-cellular biofilaments, play a significant role in many cell functions including cellular morphology, cell division, and cellular transportation. These biofilaments that are composed of alpha and beta tubulin exhibit dynamic instability, an unstable behavior that they express between two phases of growth and shortening during their polymerization [1], [2], [3], [4], [5]. Microtubules interact with microtubule-associated proteins. Besides these interactions, they are known as good targets for several antimitotic agents, such as Taxol, due to the influence of these drugs on microtubule polymerization specifications. Taxol, a member of the microtubule-stabilizing agents, increases the polymerization of microtubules and kinetically stabilizes them [6].

Among diverse microtubules-associated proteins (MAPs), Tau protein is identified as one of the major MAPs mainly found in neuronal cells. Extensive research has been conducted and reported on the reactions of Tau proteins with neuronal microtubules, both *in vivo* and *in vitro* [7], [8], [9]. Many reported results consistently identified the modulatory role of the Tau protein in polymerization of brain microtubules – findings confirm that Tau protein promotes the nucleation and the assembly of brain microtubules [10]. Other evidence indicates that the Tau protein regulates the interaction of some molecular motors and their transport along microtubules [11]. These dual interactions with microtubules and molecular motors, place Tau protein at the focus of many investigations, as their abnormal functionalities may lead to neurodegeneration diseases, such as Alzheimer and Down Syndrome [12].

While Tau proteins are known as one of the neuronal MAPs, they are observed to be expressed in several other cells, including both cancerous and normal ovarian and breast cells [13], [14], [15]. Since a possible link has been reported between the overexpression of Tau protein and drug resistance, the study of the Tau on cancer microtubules will contribute to new knowledge that may be significant in overcoming drug resistance, leading to impactful therapeutic strategies. Previous studies have found that the Tau protein interacts with the beta tubulin in the microtubules’ structure [13]. Remarkably, there are multiple different beta tubulin isotypes. Distinct from neuronal cells, such as brain cells, that consist of 3% βI, 58% β II, 25% β III, and 13% β VI, MCF7 microtubules structured from different distribution of beta tubulin isotypes: 0% β II, 39.1% of β I, 2.5% of β III, and 58.4% of β IV [16], [17], [18], [19], [20], [21], [22], [23], [24].

There is significant evidence indicating a relationship between tubulin isotypes and the intrinsic dynamic behavior of microtubules, their electro-statistical specifications, as well as the role that they play in the functions of some molecular motor along microtubules [25], [35]. Despite the accumulating reports that point to the crucial role of beta tubulin isotypes, it is still poorly understood if MAPs may differently effect polymerization of microtubules constructed with diverse distributions of isotypes. To address this, in a series of experiments *in vitro*, we studied the polymerization of MCF7 and Tau-MCF7 microtubules under well-controlled experimental conditions. We then compared the results with those obtained from our control experiment achieved using the polymerization of porcine brain and Tau-porcine brain experiments. To obtain a better understanding of biophysical differences, we then measured one of the electrostatic properties of MCF7 microtubules, in the presence and absence of Tau protein, and compared the results with each other.

## 2 Materials and methods

### 2.1 Protein preparation

We polymerized microtubules from tubulin and tubulin mixed with Tau proteins *in vitro* through the following procedure: We used two types of tubulin a) porcine brain tubulin (Cytoskeleton, Denver, Co, Cat. TL240-A) and b) MCF7 tubulin (Cytoskeleton, Denver, Co, Cat. H005). For Tau proteins, we used proteins obtained from the bovine brain source (Cytoskeleton, Denver, Co, Cat. TA01). The molecular weight of Tau proteins is between 40 and 70 kDa (as reflected in the product's datasheet) with an average of 55 kDa which is similar to the tubulin's molecular weight.

The lyophilized porcine brain and MCF7 tubulins were resuspended to 5 mg/ml in polymerization buffer (BRB80: 80 mM PIPES, pH 7.0, 0.5 mMEGTA, and 1 mM MgCl_2_ and 1.0 mM GTP). Tau proteins were resuspended to 1 mg/ml in the polymerization buffer. To polymerize Tau-microtubules, we mixed resuspended Tau proteins with tubulin (40% Tau proteins and 60% tubulin). Therefore, the concentration of tubulin was decreased to 3 mg/ml and Tau proteins to 0.4 mg/ml (7.3 µM) respectively. To conduct experiments under similar experimental conditions, other samples of tubulins without Tau proteins were diluted by the polymerization buffer to reduce the tubulin concentration to 3 mg/ml.

To polymerize microtubules, samples of porcine tubulin with and without Tau proteins were separately incubated for 5 min at 37 °C. However, due to the slow polymerization of MCF7 tubulin, samples of MCF7 and Tau-MCF7 tubulin were incubated at 37 °C for 2 h. After this incubation time, some samples of MCF7 microtubules with and without Tau proteins remained at room temperature for an extra 4 h (early visualized microtubules) while other samples remained for 6 h (delayed visualized microtubules).

In this study, we considered the polymerization of the porcine brain tubulin with and without Tau proteins as the control experiment. The goal was to verify that Tau proteins were active, and the results obtained from their polymerizations were consistent with previously reported studies.

To evaluate polymerized microtubules, samples of porcine brain, Tau porcine brain, MCF7, and Tau-MCF7 microtubules were constructed by adding a 1–1.2 µl of microtubules on a microscope slide, covered by a clean coverslip (Tedpella, thickness No. 0), sealed. Each sample was then consistently visualized in a 15–20-min time frame. A coverslip (12 mm × 12 mm) on the small amount of buffer (~ 1 µl) containing polymerized microtubules, produces surface pressure. This pressure along with sealing the sample prevents flow of microtubules in the samples and they remain immobilized during measurements. This way we could analyze the spontaneous polymerization of microtubules. However, to re-assure that no movement occurs at the time of measurements, we also implemented a method that we had previously used to measure the dynamic behavior of individual MCF7 microtubules *in vitro* from axoneme [33].

To do this, we washed individual microscope slides with axonemes (A gift from Gross's laboratory, prepared from Sea Urchin sperm. We referred the axonemes as seeds). The Tau-MCF7 and MCF7 tubulin were then added to each individual microscope slide, sealed, and incubated. Axonemes stick to the glass surface and tubulin subunits attach to the axonemes without adhering to the glass surface [4]. Each sample was visualized in a specific time frame. This way we could analyze the polymerization of microtubules from immobilized seeds. This method was also implemented here for a re-confirmation of our measurements.

### 2.2 Visualization, measurement, and analysis

Microtubules were visualized by Nikon upright microscopy (ECLIPSE- Ci-S/Ci-L), equipped with a 100 × /1.25 NA oil immersion objective lens, and a 1.43–1.20 oil dark-field condenser. The microscope was connected to a Lumenera camera (Infinity HD). The recorded videos or snapshots obtained from the field of view were analyzed by ImageJ (Rasband, W. S, ImageJ, National Institutes of Health, Bethesda, MD, USA).

In our study, we first measured the length of the selected MCF7 and porcine microtubules with and without Tau proteins. The length distribution histograms of microtubules from the collected data set were then constructed. The difference between two data sets were statistically analyzed by the Kolmogorov-Smirnov test (KS-test).

### 2.3 Calculation of microtubule polarization by electro-orientation model

To assess the dielectric specifications of Tau-MCF7 and MCF7 microtubules, we used the electro-orientation method that we recently used on MCF7 microtubules. In this method, monitoring the re-orientation of microtubules inside a uniform electric field leads to calculating the polarization factor of individual microtubules.

The uniform electric field in the experiment is produced by a micron-sized capacitor built on single microscope slides. The microscope slides were coated with Indium-Tin Oxide (with 1500 Å thickness and the ITO sheet resistance ~14ops) with an uncoated gap of 150 µm in the center. To transfer polymerized microtubules to the gap between the capacitor plates, 1.5 µl of polymerized Tau-MCF7 or MCF7 microtubules was applied in the space between two electrodes, covered by cover slips with dimensions of 12 mm × 12 mm, and consequently sealed. Each constructed electro-orientation chamber containing polymerized Tau-microtubules or microtubules in the absence of Tau protein was then placed on the microscope stage. A uniform electric field was implemented by connecting the capacitor to a DC power supply. To calculate the polarization coefficient of the individual microtubule, we measured the time variation of the microtubule's angle with respect to the direction of the field.

The torque induced on microtubule filaments by the electric field was the source of the re-orientation of microtubules. The angle θ is the orientation of the microtubule with respect to the direction of the uniform electric field, E, produced inside the capacitor. Each microtubule experiences a torque which is equal to Te=12Re[P×E], where P is the induced dipole moment and Re[]expresses the real part. The perpendicular component of the torque to the electric field which causes the re-orientation of microtubules is equal to $T_{\mathrm{ez}}=\frac{1}{2}\alpha E^{2}\mathrm{Cos}\theta \mathrm{Sin}\theta$. In this equation, α is the effective polarization coefficient. The individual microtubules also experience the viscous counter torque of $T_{v}=\pi l^{3}\eta C_{r}\frac{d\theta }{\mathrm{dt}}$, where η is the medium's viscosity and C_r_ is the form factor. The form factor for l≫r can be expressed as $C_{r}=\frac{1}{3(\ln(\frac{l}{r})-\frac{1}{2})}$. Since our experimental conditions, including the chambers’ depth and polymerization buffer were similar to the study of Minoura et al. on measuring dielectric specifications of bovine brain microtubules, we adopted the $\eta =10^{-3}$ Pa·s [36]. Knowing that the microtubule's radius is r = 12.5 nm, by measuring the length of the selected microtubules inside the re-orientation chambers, the form factor C_r_ can be calculated for every individual microtubule.

The two torques, produced by the electric field and viscosity, are balanced with one another leading to $\tau \frac{d\theta }{\mathrm{dt}}=-\mathrm{Cos}\theta \times \mathrm{Sin}\theta$, where $\tau =2\pi l^{2}\eta C_{r}/\;(\alpha /l)\;E^{2}$, known as the time constant of the orientation. The solution of this recent equation is $\ln(\tan\theta )=\ln({\tan\theta }_{0})-\frac{t}{\tau }$, where θ is the orientation of microtubules at time t and $\theta_{0}$ is the initial orientation of microtubules at t=0. The time constant, τ, can be obtained by calculating the slope of the graph, ln(tanθ) *vs.* time, for each individual microtubule. The effective polarization coefficient of microtubules normalized by their length can be consequently calculated by $\tau =2\pi l^{2}\eta C_{r}/\;(\alpha /l)\;E^{2}\;.$

## 3 Results

The major goal of our study was to evaluate and better understand the *in vitro* effect of Tau proteins when they interact with MCF7 tubulin, and consequently their polymerization behavior. Additionally, we investigated possible underlying mechanisms of the observed behavior.

As a control experiment and to assure the activity of Tau-proteins, in a set of parallel experiments, we started with polymerization of porcine brain tubulin with and without Tau proteins. The length of microtubules and Tau-microtubules was then measured, and the histograms of their lengths were constructed. The average length for the microtubules polymerized from porcine brain was obtained to be 16.46 + /-0.21 µm (mean+/-SEM, n = 45 measurements). This length for microtubules polymerized from Tau-porcine tubulin under the same experiment condition was equal to 21.24 + /-1.87 (mean+/-SEM, n = 46 measurements). The result obtained from this control experiment confirmed that Tau proteins were active. In addition, the longer average length of Tau-porcine microtubules confirmed that the polymerization of porcine brain tubulin was promoted due to the interactions with Tau proteins – an outcome which is consistent with previously reported results [9], [10], [11], [12].

**Fig. 1:**
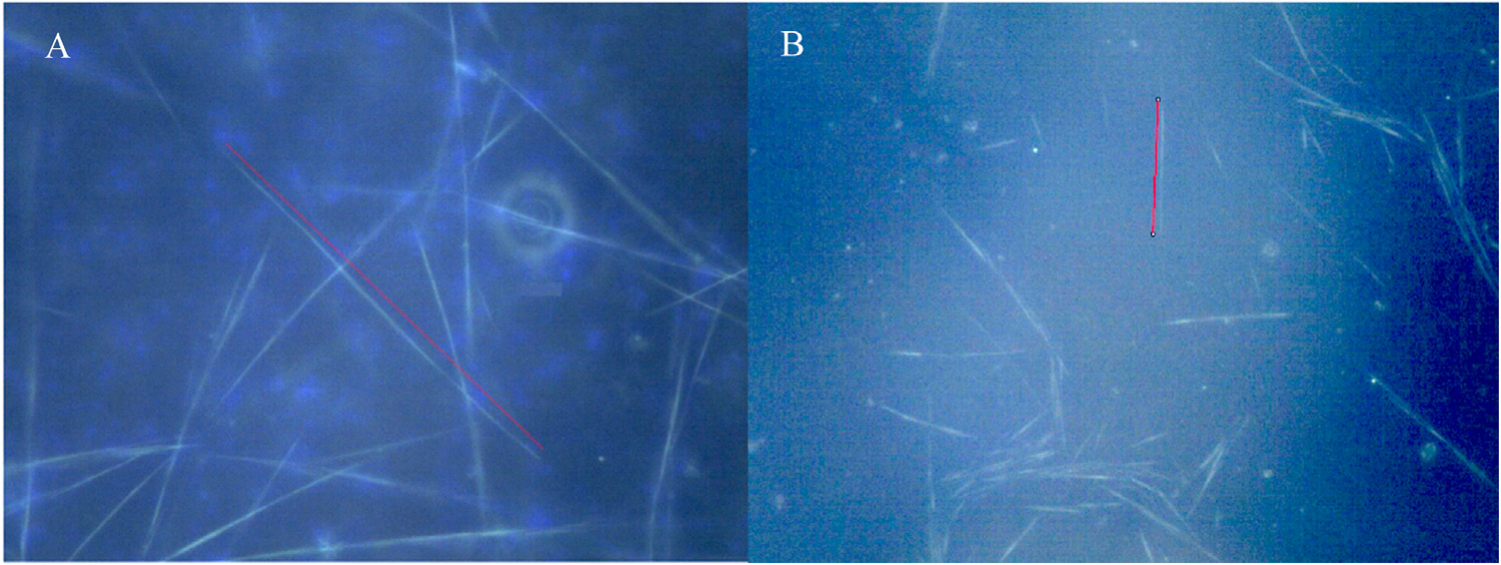
A microscope view of A) MCF 7 microtubules B) Tau-MCF7 microtubules observed under darkfield microscopy. A sample length for MCF7 is 82.95 µm (left) and for Tau-MCF7 is 18.89 µm (right).

To evaluate the effect of Tau proteins on the spontaneous polymerization of MCF7 tubulin, we polymerized microtubules from Tau-MCF7 tubulin and compared their lengths with those obtained from MCF7 tubulin in the absence of Tau proteins (Fig. 1).

**Fig. 2:**
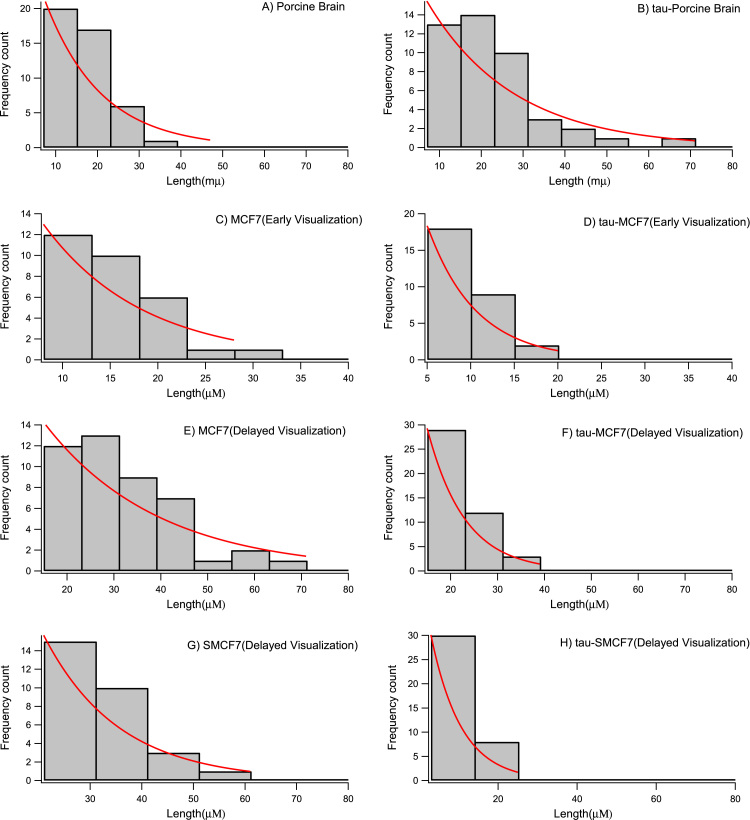
Histograms of the length distributions of A) Porcine brain: the average length was obtained to be 16.46 + /- 0.21 µm, n = 45 measurements. B) Tau-porcine brain with the average length of 21.24 + /- 1.87 µm, n = 46 measurements. The distributions are significantly different as determined by KS test. C) MCF7 early visualization with 15.41 + /- 0.87 µm, n = 29 measurements. D) Tau-MCF 7 early visualization with 9.63 + /- 0.58 µm, n = 29 measurements. The distributions are significantly different as determined by KS test. The maximum difference between the cumulative distributions D= 0.63 and D_critical_= 0.25 (E) MCF7 late visualization with 32.4 + /- 1.84 µm, n = 45 measurements. F) Tau-MCF7 late visualization with 20.69 + /- 0.88 µm, n = 50 measurements. The distributions are significantly different as determined by KS test. The maximum difference between the cumulative distributions D= 0.48 and D_critical_= 0.2. For G) MCF7-seeds late visualization, the average length was obtained to be 30.26 + /- 1.6 µm, n = 34 measurements and H) the average length for Tau-MCF7-seeds was 11.78 + /− 0.63, n = 38 measurements. These distributions are significantly different as well as determined by KS test. The maximum difference between the cumulative distributions D= 0.85 and D_critical_= 0.22. The length distributions were fitted with exponentially decaying function, consistent with other reported studies [44].

As we previously reported MCF7 are slow growing microtubules [33]. Therefore, they were visualized after a long incubation time. For the first group, which were visualized earlier, the average length was 15.41 + /-0.87 for MCF7 (mean+/-SEM, n = 29 measurements) and 9.63 + /-0.58 for Tau-MCF7 (mean+/-SEM, n = 29 measurements). For the samples visualized with a delay, the measured average length for MCF7 was 32.4 + /-1.84 (mean+/-SEM, n = 45 measurements) and for Tau-MCF7 was 20.69 + /-0.88 (mean+/-SEM, n = 50 measurements). We also repeated the experiment when microtubules were polymerized from axonemes (seeds). The samples were visualized with a delay. The average length for seed-connected MCF7 microtubules was 30.26 + /-1.6 (mean+/-SEM, n = 34 measurements). This measurement for the seed connected Tau-MCF7 was 11.78 + /-0.63 (mean+/-SEM, n = 38 measurements). The average lengths were statistically significantly different (Fig. 2).

**Fig. 3:**
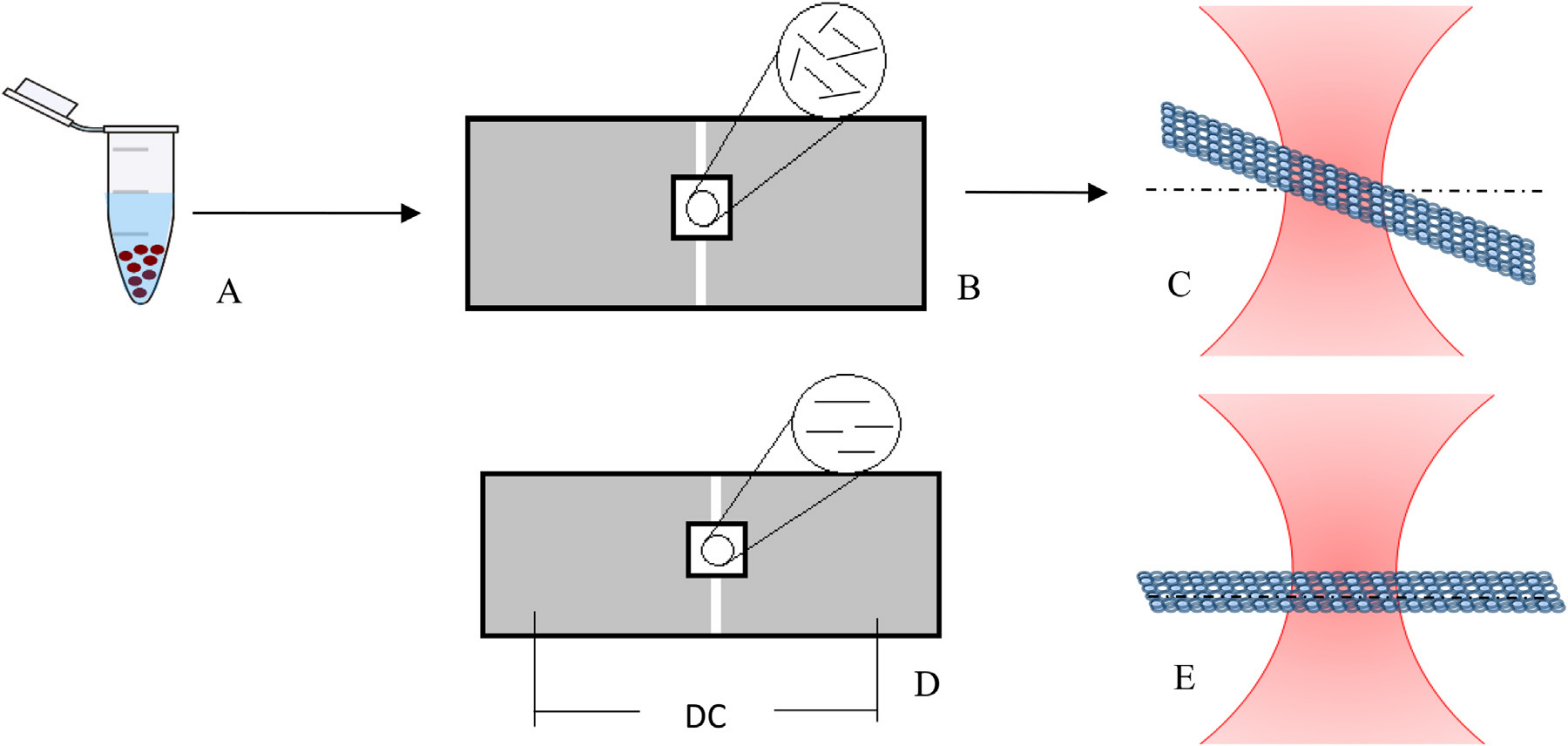
Schematic view of experimental procedure. A) Polymerized microtubules in the presence of Tau proteins are placed between the plates of the micro-capacitor. B) A micro-capacitor is built on the surface of the microscope slide. After transferring microtubules by placing and sealing a cover slip, a reorientation chamber is built. Microtubules in this chamber are randomly oriented. C) Microtubules will be visualized under darkfield microscopy. D) A uniform electric field is produced by connecting the plates to a DC power supply. E) As a result of this electric field, microtubules are reoriented and become parallel with the direction of the electric field. The time variation of the angle between individual microtubules and the direction of the electric field are measured.

In contrast with the promoted polymerization of Tau brain microtubules, a demoted polymerization was observed in Tau-MCF7 microtubules. We investigated the roots of this difference in possible distinction that may exist between dielectric specifications of MCF7 and Tau-MCF7 microtubules. To test this, we calculated the polarization coefficient of Tau-MCF7 and MCF7 microtubules by monitoring their re-orientation behavior in a uniform electric field and compared the result with one another as well as our recently reported findings obtained from MCF7 microtubules [35]. (Fig. 3)

As explained in the previous section, the re-orientations of Tau-MCF7 microtubules with respect to the uniform electric field were monitored and recorded. The minimum electric field of 0.21 × 10^-5^ V/m (while the implemented voltage was 4 V, the reading voltage across the electrode was measured to be 3.16 V) was implemented. The re-orientation of microtubules and their alignments with the direction of the electric field were observed. Using the Image J software, the time variation of the angle of individual microtubules with respect to the direction of the electric field was measured. By calculating the inverse of the slope of the linear fit to the data plotted in the Lntan(θ) *vs.* time graph, time constant, τ, and consequently, the normalized polarization coefficient over the length of microtubule, α/l, was calculated using $\tau =2\pi l^{2}\eta C_{r}/\;(\alpha /l)\;E^{2}$. The re-orientation behavior of 8 Tau-MCF7 microtubules with the length between 15 and 23 µm were monitored inside the 0.21 × 10^-5^ V/m electric field. The average value for the normalized polarization coefficient was obtained to be 1.2 + /-0.2 × 10^–22^ Cm/V. We repeated the experiment under the same conditions for MCF7 microtubules. 4 MCF7 microtubules between 17 and 26 µm were assessed. The obtained value was 4.9 + /-0.6 × 10^–22^ Cm/V. This higher value of the normalized polarization coefficient for MCF7 microtubule is consistent with previous measurements [35]. These two averages are statistically significantly different as determined by the *t*-test (P value is less than 0.0001). These two values are compared in Table 1.

**Table 1:** A comparison between normalized polarization coefficient, α/L, of MCF7 microtubules with and without Tau proteins.

Polymerized Microtubules (MT)	α/L
Tau-MCF7	1.2 + /− 0.2 × 10^–22^ Cm/V
MCF7	4.9 + /− 0.6 × 10^–22^ Cm/V

## 4 Discussion

Many regulatory factors in polymerization and dynamic behavior of microtubules include, but are not limited to, the interactions with microtubule-associated proteins, anticancer agents, or even interactions with some molecular motors [9], [10], [11], [12], [13]. In addition, the results of our studies and others indicate that the differences which exist in the composition of microtubules in terms of distribution of beta tubulin isotypes, may also alter both their biomechanical specifications, as well as functions of some molecular motors along them [25], [26], [27], [28], [29], [30], [31], [32], [33], [34], [35].

One of the microtubules associated proteins (MAPs), Tau proteins that are known as neuronal MAPs contribute to the stability of neuronal microtubules. However, Tau overexpression observed in cancerous breast and ovarian cells is linked to the level of resistance they display while interacting with Taxol. For instance, in the study by Rouzier et al., the stabilizing effect of Taxol on microtubules inside some MCF7 cells has been reduced, which has been associated to the overexpression of the Tau protein in these cells [14], [15]. In contrast, other reported studies suggest less of a correlation between Taxol resistance and Tau overexpression [37].

In our study, we directly tested the effect of Tau proteins on MCF7 microtubules *in vitro* and in the absence of other cellular regulatory factors. The shorter value obtained for the average length of Tau-MCF7 *versus* MCF7 microtubules is intriguing.

On one hand, we know that the Tau proteins interact with beta tubulin, but, on the other hand, the diversity of beta tubulin isotype distribution in microtubules’ structure of different cells make them distinct from one another. Considering these two fundamental factors, the demoted polymerization of Tau-MCF7 observed in this study suggests that the regulatory effect of Tau proteins on microtubules polymerization behavior can potentially be beta-isotype-specific and be altered in different cells.

From an electrostatic perspective, the reduced value obtained for the normalized polarization coefficient, and therefore, the charge on Tau-MCF7 microtubules can possibly change the affinity and binding of different beta tubulin isotypes that, in fact, carry different amount of charges in their C-terminal domains.

Additionally, there is some evidence indicating that the Taxol resistance in breast cancer cells may be the result of an increase in the level βIII isotypes [38], [39], [40], [41], [42], [43]. Whether the change of dielectric specifications of microtubules caused by Tau proteins can selectively attract a specific type of beta isotypes, namely βIII, and consequently change the stability of cancer microtubules is currently an open question. While the growing evidence supports the critical role of beta tubulin isotypes in the way that microtubules interact with other MAPs or chemotherapeutic drugs, identifying the factors that can be responsible for the underlying mechanisms is imperative for our knowledge of Cancer Biology, with the hope of developing more effective treatments.
