# Supplementary material for: The regulatory effect of Tau protein on polymerization of MCF7 microtubules in vitro
Source: Biochem Biophys Rep. 2019 Jan 9;17:151–6. doi: 10.1016/j.bbrep.2018.12.010 (PMC6327910; doi:10.1016/j.bbrep.2018.12.010)
Supplement: Supplementary file 1 — Supplementary material [file mmc1.docx]

The authors report no conflicts of interest related to this study.
